# Zootoxins and Domestic Animals: A European View

**DOI:** 10.3390/toxins16010048

**Published:** 2024-01-16

**Authors:** Andras-Laszlo Nagy, Sabrina Ardelean, Ronan J. J. Chapuis, Juliette Bouillon, Dalma Pivariu, Beatrice De Felice, Mirko Bertazzo, Paola Fossati, Leon J. Spicer, Alexandra Iulia Dreanca, Francesca Caloni

**Affiliations:** 1 Department of Biomedical Sciences, Ross University School of Veterinary Medicine, Basseterre P.O. Box 334, Saint Kitts and Nevis; anagy@rossvet.edu.kn (A.-L.N.); rchapuis@rossvet.edu.kn (R.J.J.C.); 2 Department of Clinical Sciences, Ross University School of Veterinary Medicine, Basseterre P.O. Box 334, Saint Kitts and Nevis; aardelean@rossvet.edu.kn (S.A.); jbouillon@rossvet.edu.kn (J.B.); 3 Department of Toxicology, Faculty of Veterinary Medicine, University of Agricultural Sciences and Veterinary Medicine Cluj-Napoca, Calea Manastur 3-5, 400372 Cluj-Napoca, Romania; dalma.pivariu@usamvcluj.ro (D.P.); alexandra.dreanca@usamvcluj.ro (A.I.D.); 4 Department of Environmental Science and Policy (ESP), Università degli Studi di Milano, Via Celoria 10, 20133 Milan, Italy; beatrice.defelice@unimi.it (B.D.F.); mirkobtz@gmail.com (M.B.); paola.fossati@unimi.it (P.F.); 5 Department of Animal and Food Sciences, Oklahoma State University, Stillwater, OK 74078, USA; leon.spicer@okstate.edu

**Keywords:** domestic animals, marine toxins, terrestrial toxins, toxicity, zootoxicosis, zootoxins

## Abstract

Zootoxins are produced by venomous and poisonous species and are an important cause of poisoning in companion animals and livestock in Europe. Little information about the incidence of zootoxin poisoning is available in Europe, with only a few case reports and review papers being published. This review presents the most important zootoxins produced by European venomous and poisonous animal species responsible for poisoning episodes in companion animals and livestock. The main zootoxin-producing animal species, components of the toxins/venoms and their clinical effects are presented. The most common zootoxicoses involve terrestrial zootoxins excreted by the common toad, the fire salamander, the pine processionary caterpillar, and vipers. The lack of a centralized reporting/poison control system in Europe makes the evaluation of the epidemiology of zootoxin-induced poisonings extremely difficult. Even if there are many anecdotal reports in the veterinary community about the exposure of domestic animals to terrestrial and marine zootoxins, the number of published papers regarding these toxicoses is low. Climate change and its consequences regarding species distribution and human-mediated transportation are responsible for the emerging nature of some intoxications in which zootoxins are involved. Although new venomous or poisonous animal species have emerged in regions where they were previously unreported, zootoxins produced by native species remain the main concern in Europe. The diversity of poisonous and venomous animal species and the emerging nature of certain poisonings warrant the continuous update to such knowledge by veterinary professionals and animal owners. This review offers an overview about zootoxin-related poisonings in domestic animals in Europe and also provides important information from a health perspective.

## 1. Introduction

An aspect that is under investigated, considering the European context when compared with other geographical areas [1,2,3], is the exposure of domestic animals to natural toxins of animal origin, commonly classified as marine and terrestrial zootoxins (Table 1 and Table 2). Zootoxins, produced by poisonous/venomous animals (Table 1) [2], are generally a mixture of different chemicals with species-specific toxicological effects on animals, which are related to many factors including age, sex, and physiological conditions [2]. Zootoxin exposure is not very frequent and is considered relatively rare [2], as shown by numerous and, even, recent European toxico-epidemiological studies on poisoning in animals [4,5,6], representing around 2–5% of total poisonings [6]. Moreover, there is an ever-growing interest from one health perspective that considers animals as sentinels of novel emerging zootoxicoses (Table 3), as well as in correlation with climate change and its impact on venomous terrestrial and marine animal species and zootoxin distribution [6,7,8]. The available data and case reports indicate that mainly native species are involved in zootoxicoses in Europe. In the future, species redistribution may lead to a predominance of invasive species, so careful monitorization of emerging toxicoses is necessary to implement effective diagnostic, preventive, and therapeutic methods.

The most common animal sources of terrestrial zootoxins involved in zootoxicosis in domestic animals are: the common toad, *Bufo bufo* [6,9], the fire salamander, *Salamandra salamandra* [6,10], the pine processionary caterpillar, *Thaumetopoea pityocampa* [4,5,6], and venomous vipers, *Vipera aspis aspis* and *Vipera berus berus* [5,6,11,12]. The exposure of domestic animals to marine toxins from dinoflagellates or algae (domoic acid, gonyautoxins, ciguatera), cnidarian toxins (anemones, jelly fish, etc.), echinoderm toxins (sea stars, starfish, etc.), mollusk toxins (cone shells, octopus, etc.), or fish toxins (venoms and poisons), is very rare [6,13]. Recently in the UK, case reports involving dogs related to their exposure to paralytic shellfish toxins [13], palytoxin [13], and jelly fish (phylum Cnidaria), were collected [14]. Jellyfish poisoning was recently reported in Italy, as well [6]. The aim of this review is to provide a complete overview of the European situation in regard to zootoxins and domestic animal exposure, as well as provide an analysis of emerging case reports.

Terms used to identify relevant publications in academic databases (Google Scholar, Web of Science Core Collection, Scopus, MEDLINE/PubMed) were “zootoxin”, “blister beetle”, “Meloidae”, “cantharidin”, “honeybee”, “melittin”, “wasp”, “caterpillar”, “thaumetopoein”, “centipede”, “Scolopendra”, “Fire salamander”, “samandarin”, “marine toxins”, “Jellyfish”, “cyanobacteria”, “blue-green algae”, “paralytic shellfish toxins”, “palytoxin”, “sawfly”, “Arge pullata”, “scorpion”, “scorpion neurotoxin”, “snake envenomation”, “snakebite”, “Viperidae”, “Vipera berus”, “Vipera ammodytes”, “tick paralysis”, “toad”, “bufo”, “bufodienolides”. Searches using associations between the mentioned keywords and “Europe”, “animals”, “veterinary”, or “toxicosis” were also performed. Book chapters in veterinary toxicology reference books were reviewed for relevant information.

## 2. Toxic and Venomous Species Involved in Domestic Animal Poisonings

### 2.1. Blister Beetles

Among the more than 300,000 species of beetles described worldwide, less than 100 are considered of veterinary or public health importance [2]. The most important beetles for human health and veterinary medicine are members of the *Meloidae* family (blister beetles). Blister beetles are insects with a soft, elongated body, and a narrower pronotum than the head or wings [2].

Adult blister beetles are often brightly colored, the need for camouflage being eliminated by their ability to secrete cantharidin [15], a toxic colorless substance. It is soluble in organic solvents and, a monoterpene anhydride compound with vesicant properties [2,16,17], the latter of which when in contact with a mucosal surface, results in acantholysis and vesicle formation leading to ulcers or erosions [17]. Cantharidin is produced by the insects to protect their eggs, and the quantity produced depends on the species, sex (males have higher concentrations), season, and available food source [16]. Cantharidin is an inhibitor of the serine–threonine protein phosphatase [18], disrupting signal transduction and cell metabolism [2]. The primarily affected organelles are the mitochondria with active transport across the mitochondrial membrane being affected via consecutive permeability changes, cellular disruption, and acantholysis [2]. Cantharidin also blocks adenosine A1 receptors, plays an important role in the regulation of myocardial oxygen consumption, and induces antiadrenergic effects in ventricular cardiac muscle cells [19]. Thus, indicating the heart as a target organ of cantharidin. The clinical signs induced by exposure to cantharidin are represented by intense irritation of the skin and the esophageal, gastric, and intestinal mucous membranes [2]. After systemic absorption, the irritant effect is exerted on the urinary tract, especially the bladder and urethra. The consequence of the irritant effect is gastroenteritis, cystitis, and/or urethritis and nephrosis [2]. In horses, the median lethal dose of cantharidin is 1 mg/kg, while in cats and dogs it is 1–1.5 mg/kg [19]. Massive doses of cantharidin may cause shock and death within 4 h.

The cardiac symptoms include tachycardia and myocardial dysfunction [19]. The positive inotropic effect is due to increased calcium influx in cardiomyocytes. Cardiac signs are associated with congested mucous membranes and decreased capillary refill time [2]. Pulmonary edema is frequently observed in affected animals [2].

The most common gross lesions induced by cantharidin are oral and esophageal ulcers, ulcers of the stomach and intestines, hemorrhages in the renal cortex, and hemorrhages in the urethra and urinary bladder. In the heart, the main lesion is ventricular myocarditis. Splenomegaly, hepatomegaly, and pulmonary edema are also observed [2]. Microscopic lesions are represented by acantholysis of the gastrointestinal mucosa, vascular endothelium, and urinary tract epithelium [2].

Most of the cantharidin poisoning cases in animals are reported in horses in North America [2,16,17]. The most important blister beetle species involved are the striped blister beetle (*Epicauta vittata*), the black blister beetle (*Epicauta pennsylvanica*), the margined blister beetle (*Epicauta pestifera*), and the three-striped blister beetle (*Epicauta lemniscata*) [2]. These North American species feed on flowering foliage, especially blooming alfalfa. Modern harvesting methods, like crimping, increase the incidence of the disease by increasing the number of these beetles trapped in hay [2,16].

Scarce information is available in Europe about cantharidin poisoning in animals. Recently, possible cantharidin poisoning cases in horses were reported in the Mediterranean part of Slovenia [16]. In these cases, the diagnosis was based on the identification of insect parts in the stomach contents collected after gastric lavage, and in the freshly cut grass fed to the animals [16]. In the area where these cases were reported, Jakovac and coworkers performed entomological surveillance [16], and *Mylabris variabilis*, as well as *Epicauta rufidorsum,* were identified as the most abundant blister beetle species [16]. *Mylabris variabilis* is native to Southern Europe, from the Iberian Peninsula to southern Russia [20].

*Berberomeloe majalis* is another important blister beetle species and is found in the western Mediterranean basin, the Iberian Peninsula, southern France, and north Africa, from Morocco to Tunisia [21]. Recently, in Spain, a suspected case of cantharidin poisoning was reported in a great bustard (*Otis tarda*) [2,22]. The blister beetle, *Berberomeloe majalis,* was involved in this case [22].

Another important European blister beetle species is *Lytta vesicatoria*, commonly called the Spanish fly [15], which was historically an important source of cantharidin, used as an aphrodisiac by humans [23]. Accidental ingestion of these preparations can potentially result in toxicosis in animals.

### 2.2. Bees and Wasps

Stings by insects are a common problem in domestic animals and are encountered worldwide [2]. Among these insects, the European honeybee (*Apis mellifera*) is the most common species of bees involved in stings and envenomation [2,24,25]. Despite its global occurrence, only a few case reports describing bee envenomation in domestic animals exist [25]. Although the European honeybee is involved in most of the cases, the Africanized honeybee is more aggressive, and the likelihood of multiple stings by a swarm is greater [2,26].

Vespid wasps (family: *Vespidae*) have worldwide distribution and comprise thousands of species [27]. In Europe, the most prevalent species is the European wasp or German wasp (*Vespula germanica*) [28]. Another important species present in Europe is the common wasp (*Vespula vulgaris*) [29]. These insects have a special venom delivery system, which consist of modified ovipositor apparatus and is found only in female bees and wasps [2]. The stinger of the honeybee is covered with barbs that cause the stinger to remain in the wound and continue to release toxins, while the stinger of wasps is smooth [2,30]. Bees usually do not sting unless they are provoked [25].

Honeybee venom is a transparent acidic substance, consisting of a complex mixture of proteins, peptides, enzymes, and small molecules, including melittin, phospholipase A2, and hyaluronidase, which are the main constituents responsible for the allergic reactions in exposed animals [2,25]. Phospholipase A2 is the most toxic component of the venom. Melittin is a disruptive transmembrane protein that enhances the damage caused by phospholipases [2,31]. In addition, melittin causes pain, triggers hemolysis, and increases blood flow and cell permeability, enhancing the spread of venom in tissues [2]. Melittin, together with phospholipase and the mast cell degranulating peptide (a cationic 22-amino acid residue peptide), stimulates mast cell degranulation and triggers the release of histamine and serotonin [2,32]. Melittin and phospholipase A2 are able to induce hemolysis (by inducing the formation of transient pores in red cell membranes, with consecutive leakage of hemoglobin molecules) in domestic animals and, at low doses, induce echinocyte formation and, at high doses, induce spherocyte formation [31]. Another component of bee venom is apamin. Apamin is a neurotoxin that blocks calcium-activated potassium channels, resulting in transient peripheral nerve effects [2].

Wasp venom contains peptides (mastoparan, eumenitin, eumenitin-R, rumenitin-F, EpVP, decoralin, and anoplin), enzymes (hyaluronidase, α-glucosidase, phosphatase phospholipase A2, and phospholipase B), and amines (serotonin, histamine, tyramine, and catecholamines) capable of triggering pain [2,27]. Other active ingredients are kinins and acetylcholine [2]. Kinins are the primary components responsible for the pain induced [2]. All these components contribute to local vasoactivity and the pain induced by wasp venom [2]. Many of the venom components can trigger allergic reactions [2].

Honeybees can only inflict a single sting, because the stinger remains in the wound, but the victim may sustain multiple stings if they are attacked by a large number of bees [2]. In these cases, the cumulative effect of multiple envenomation may be lethal [2]. The stinger of wasps is smooth and, consequently, a single wasp may inflict multiple stings. Also, wasps are highly social, which further increases the likelihood of multiple stings [2]. In most animal species, the lethal dose of bee/wasp sting is approximately 20 stings/kg, but the anaphylactic reaction is not dose dependent [26]. The European honeybee sting delivers approximately 147 μg venom, while a wasp sting delivers approximately 17 μg of venom [30].

The severity of the clinical signs depends on whether the animal was stung once or multiple times. A single bee or wasp sting results in local reactions, consisting of edema, swelling, pain, and erythematous plaques at the site of the sting [2,26]. Dogs and cats frequently present facial, periorbital, and/or aural edema [2]. Local signs are usually self-limiting [30]. An abscess may form around the bee stinger at the sting site [2].

Multiple stings may induce systemic effects. Anaphylactic shock is an important consequence of multiple bee or wasp stings, and most deaths are related to this immediate hypersensitivity reaction [25,26]. Ataxia and facial nerve paralysis are possible clinical signs of multiple bee stings and are caused by apamin present in the bee venom [25]. Prostration, hyperthermia, hematemesis, bloody diarrhea, intravascular hemolysis, thrombocytopenia, hematuria, and incoordination are other clinical signs associated with multiple stings [2,25]. Clinical signs in horses exposed to multiple stings are agitation, urticaria, angioedema, rhabdomyolysis, hypovolemia, ileus, and renal failure [2]. Anaphylactic shock in dogs is characterized by local upper airway edema and respiratory obstruction, cardiovascular compromise, seizures, and weakness. Urticaria, pruritus, angioedema, vomiting, and urination are also seen [30]. Anaphylactic shock in cats is characterized by pruritus, hypersalivation, ataxia, and collapse [30].

Delayed-type hypersensitivity reaction may occur 3–14 days after the sting and is characterized by disseminated intravascular coagulation, arthritis, vasculitis, neuropathy, and renal failure [30].

Lesions induced by the sting of bees and wasps are not characteristic [25]. In horses, skin lesions (e.g., urticaria, edema) are often seen, which is consistent with multiple stings. These are more difficult to detect in dogs and cats. Facial edema, periorbital edema, muscular necrosis, splenomegaly, congestion and hemorrhage in different organs, dark red urine, icterus, and hemoglobin casts inside renal tubules are possible lesions [25]. Laryngeal edema, congestion, and hemorrhage are possible in fatal cases of anaphylactic shock [25].

In recent years, only a small number of case reports in Europe concerning bee or wasp stings have been published. Several papers published in Switzerland have described the use of immunotherapy in the treatment of anaphylactic shock associated with a Hymenoptera sting in dogs [33,34], or investigate these stings as a cause of urticaria in dogs [35].

### 2.3. Caterpillars

Caterpillar envenomation is a global public health hazard for both humans and animals [36]. Belonging to the order of insects known as Lepidoptera, which includes 133 species of moths and butterflies [36,37], 9 of the 133 families are poisonous worldwide [36]. Particularly, in Central and Southern Europe, two species, pine (*Thaumetopoea pityocampa*) and oak (*Thaumetopoea processionea*) processionary caterpillars are responsible for most human and animal envenomation [36,37,38,39,40]. Present in both urban and rural areas, the latitudinal and altitudinal geographic distribution of these species has expanded as a consequence of global warming and human-mediated transportation, causing an increased incidence of envenomation [40,41]. These insects’ life cycle lasts for about 1 year [37]. Moths typically emerge and fly between June-July and September. They live for 1–2 days and lay eggs in trees. The first instar larvae hatch around April and build silky nests in trees. The larvae feed and defoliate the trees before dropping onto the ground and crawling in procession to colonize other trees [37,42].

The processionary caterpillar’s defense mechanism involves the production of urticating setae through larval stages 3 to 5 [43]. There are approximately 1,000,000 setae (similar to hairs of 150–200 μm) per larva in *T. pityocampa*, with a sharp proximal end and pointed barbs directed distally [37,42]. Setae are not innervated and can become airborne after being actively released by the larvae when disturbed [38,42]. Airborne setae may disperse over long distances (several kilometers) and can persist for a long time in the environment (up to 12 years) [37]. Setae cause toxicosis through a dual mechanical-toxic effect. First, the hair causes skin lesions through the mechanical action of penetration through the skin. Second, the distal aspect of the setae, containing the venom, can break, which leads to the release of various proteins causing toxic, irritant, and allergic responses [43]. Thaumetopoein, the main active compound, has demonstrated urticating properties in guinea pigs and can induce mast cell degranulation through non-immune and immune mechanisms [43,44]. Another 10 different proteins have allergenic properties, with Tha p 1 and Tha p 2 (which may be thaumetopoein) being the most common [38,44,45,46].

The spectrum of clinical signs associated with caterpillar envenomation is defined as lepidopterism [47]. Lepidopterism appears to affect mainly dogs and cats in Europe. Two retrospective studies from France report a prevalence of caterpillar envenomation of 0.13% and 0.57% in cats and dogs, respectively [48]. Most cases of envenomation are presented from January to September, when caterpillars crawl in procession on the ground [39,47,48]. It typically involves oral, cutaneous, and ocular lesions in both humans and animals [39,42,47,48]. Systemic signs may occur and may be associated with gastrointestinal and/or airway inflammation as a consequence of ingestion/inhalation of setae, or reflect systemic inflammatory response syndrome [48,49]. To the authors’ knowledge, anaphylaxis has only been described in humans, but may occur in small animals [49]. The survival rate is high (97–100%) after treatment [39,48]. Most of the lethal cases in dogs were presented in respiratory distress, with a few cases associated with upper airway obstruction, secondary to soft tissue swelling [48]. Tongue lesions and tongue necrosis were common complications in dogs, but the long-term outcome was good, and patients did not present dysphagia [47,48]. One study identified tongue color changes and hyperthermia (increased rectal temperature) as predictors of lingual necrosis; at admission, for each degree above 39.15 °C, the risk of having tongue lesions increased by 1.8-fold [48]. Tongue necrosis is less common in cats, which may be attributed to their different behavior towards caterpillars [39]. Hematological abnormalities were uncommon (4.8–14%) and mainly consisted of anemia, leukopenia, leukocytosis, and azotemia [47,48]. Pine processionary caterpillar envenomation was also reported in dogs in Italy [6]. To the authors’ knowledge, there has been no report of lepidopterism in large animals in Europe. However, in North America and Australia, exposure to eastern tent caterpillars (*Malacosoma americanum*) or bag-shelter processionary caterpillars (*Ochrogaster lunifer*) was associated with mare reproductive loss syndrome or equine amnionitis and fetal loss syndrome, respectively. The pathogenesis associated with these syndromes remains unclear, but recent studies suggest that an unidentified toxin may be responsible for inciting tissue inflammation, leading to bacterial translocation and abortion [50,51,52]. Finally, in Canada, the ingestion of forest tent caterpillars (*Malacosoma disstria*) by horses may have caused septic fibrinous pericarditis [53]. Therefore, it may be prudent to avoid the ingestion of caterpillars by horses in Europe.

### 2.4. Centipedes

Although centipedes are present worldwide, centipede envenomation is infrequently reported in humans and is not reported in animals [54]. However, the exact incidence of centipede poisoning is unknown, as many cases do not require medical evaluation [54,55,56]. Additionally, there is evidence of dog and cat interactions with centipedes, indicating their possible exposure to bites [57,58].

Centipedes belong to the phylum Arthropoda, subphylum Myriapoda, and the Chilopoda class [59]. There are approximately 3500 species of centipedes divided into five orders: Scutigeromorpha, Lithobiomorpha, Craterostigmomorpha, Geophilomorpha, and Scolopendromorpha [55]. It is estimated that only 15 (0.43%) of these species are clinically important, with the majority of these from the *Scolopendra* species [54,55]. Hundreds of species have been identified in Europe, including *Scolopendra* species [60,61,62,63,64,65,66,67,68,69,70]. Despite this, most reports in humans describe centipede envenomation in tropical or subtropical areas, as centipede bites appear to be less severe in Europe [56]. Indeed, venom toxicity varies among species [59]. Their venom contains approximately 50 peptides and proteins acting as neurotoxins, myotoxins, cardiotoxins, as well as pro- and anticoagulant toxins [55]. Centipedes have a pair of forcipules (modified front legs) that are connected to venomous glands and act as fangs [2]. The legs of *Scolopendra* spp. present sharp claws that can penetrate the skin, introducing the venom into the wound. The sting and the inflicted toxins cause irritation and inflammation due to mast cell degranulation [2]. The main clinical signs of centipede envenomation in humans include local pain, erythema, bruising, and swelling [54,55]. Although there is no report of centipede envenomation in small animals, it is commonly accepted that these animals may develop clinical signs like humans, though to a lesser extent, whether it be due to natural resistance or different behavior towards the predator. Further studies are warranted to evaluate the risk of centipede envenomation in domestic animals in Europe.

### 2.5. Fire Salamander

Poisonous amphibians are ubiquitous in Europe. Among many genera, only a few, *Salamandra* (fire and alpine salamanders), *Bufo, Bombina*, and *Pleurodeles* can have relevant toxic effects on humans and animals [10,71]. The largest species of salamander in Europe is the fire salamander (*Salamandrinae, Salamandra salamandra*) [72]. The poison glands of a fire salamander are concentrated in and around specific regions of its body, particularly the skin surface on its head and back. These glands typically mirror the animal’s skin colorations, being in the colored portions of the animal’s skin [72].

Salamandra skin poison is composed of multiple compounds [73], including different alkaloids [74,75].

Samandarine and Samandarone are the two most important alkaloid toxins produced by the poisonous glands of fire salamander (10), with samandarine serving as the primary poison [10,76]. Both are lipid-soluble steroidal alkaloids [77]. Little is known about the mechanisms of action of these alkaloids [10]. Both are irritants to the mucous membranes and have neurotoxic effects, acting mainly on the spinal cord [10]. Samandarin is the main alkaloid and is neurotoxic, causing convulsions, hypertension, and respiratory paralysis in humans and domestic animals [78].

Domestic animals are exposed to salamander poison by eating, licking, or mouthing the amphibian. Another form of exposure is the spraying of alkaloids by the amphibian from the middorsal skin glands as a defense mechanism, without the application of external pressure [10]. The clinical signs are irritation of the oral mucosa, muscle convulsions, muscle tremors, hypertension, hyperventilation, cyanosis, and cardiac arrhythmias [10,77].

Even if there are many anecdotal reports on dog poisoning involving the fire salamander, only a few case reports have been published [6,71]. A possible cause of the scarce information about fire salamander poisoning in domestic animals in the scientific literature is the acute evolution and usually fatal outcome of the disease, with many of the cases remaining undiagnosed [10].

A case of fire salamander poisoning was reported in Slovenia by Erjavec and coworkers [10], who were presented with a 12-year-old mix breed female dog, which had bitten a fire salamander about 15 min earlier. At presentation, the dog was in lateral recumbency, and showed muscle tremors, seizures, apneustic agonal breathing, and hypersalivation, with a poor response to tactile stimuli. The dog had cyanotic mucous membranes, a rectal temperature of 40.7 °C, and a pulse of 100/min. The condition of the dog rapidly deteriorated, requiring intubation and mechanical ventilation. Aggressive symptomatic and supportive therapy led to the full recovery of the dog in 4 h [10]. Between January 2015–March 2019, the Poison Control Centre of Milan (CAV) received two calls about dogs exposed to fire salamander toxins [6].

### 2.6. Marine Toxins

#### 2.6.1. Jellyfish

The group of marine organisms from the phylum Cnidaria includes anemones, stinging hydroids, sea wasps, jellyfish, and fire coral [79]. Jellyfish are invertebrate animals, with numerous long tentacles and a bell-like body [79]. On the tentacles, there are numerous nematocysts, which are stinging cells, and are used by the jellyfish to capture prey and defend themselves against predators. Nematocysts are also responsible for the clinical effects observed in animals. Millions of nematocysts can pierce the skin and inject venom upon contact with a jellyfish tentacle, resulting in discomfort and swelling. After dying, jellyfish still exhibit toxic potential because the stingers on the jellyfish can continue reacting for several weeks after the jellyfish dies [80]. The sting usually occurs in lightly furred areas, such as the face, feet, and abdomen, or in the oral cavity, if the jellyfish is put in the mouth [81]. The poison contains several bioactive compounds, and neurotoxic, cytolytic, hemolytic, and enzymatic (proteases, phospholipases) toxins, including prostaglandins (15R)-PGA2, palytoxin, pseudopterosin, sarcodictyins, and eleutherobin [6,82]. Clinical signs of jellyfish poisoning are represented by pain, oral irritation and swelling, sialorrhea, and minor gastrointestinal signs [6]. Dyspnea, hyperthermia, nausea, cardiac arrhythmias, licking the stung area, muscle cramps, and retching, are also possible signs [81,83]. Jellyfish poisoning in domestic animals is reported all over the world. In Europe, cases have been reported in the United Kingdom [14,80] and Italy [6]. In all these cases, the poisonings involved dogs, and the main clinical signs were oral irritation and minimal gastrointestinal symptoms [6,14]. In humans, jellyfish poisoning is not rare. In a review paper published in 2016, Montgomery and coworkers identified 79 publications on toxicity and treatment for human envenomation by European jellyfish species [84].

Jellyfish poisoning is an emerging disease, as climate change and its implications on venomous marine and amphibian species will have a significant impact on these poisonous animals [8]. An increase in poisonous population numbers, particularly jellyfish, and a decrease in the amphibian Bufo species is expected [8].

#### 2.6.2. Paralytic Shellfish Toxins and Palytoxin

Paralytic shellfish toxins (PSTs) are produced by prokaryotic freshwater cyanobacteria and eukaryotic marine dinoflagellates. PSTs are a group of toxins that cause a disease called paralytic shellfish poisoning (PSP), through the blockage of voltage-gated sodium channels [85].

It is known that a wide range of shellfish, such as clams, mussels, geoducks, oysters, and snails, can become contaminated with PSTs due to the filtering activity of shellfish and, therefore, can accumulate the toxins in their tissues. To date, there is no information on crab meat contaminated by toxins. However, it is possible that the gut may present toxic levels of PSTs after the ingestion of cyanobacteria or dinoflagellates. Initially, PSTs were generally called saxitoxins, since the first PST that was characterized was the saxitoxin itself. However, nowadays, PSTs are a large group of more than fifty toxins, which includes saxitoxin (STX) and several of its derivatives, formed by the addition of sulfo, hydrosulfate, and N-1-hydroxyl groups [86]. Saxitoxins and saxitoxin analogs have a common mechanism of action, namely they block voltage-gated sodium channels, leading to neuromuscular weakness [85,86].

Although it is not common for companion animals to be exposed to marine toxins, recently some case studies have occurred involving dogs. Between 2017 and 2018, in England, there were multiple incidents of illness in dogs reported, due to the consumption of marine animals. The dogs were reported to have eaten different beached marine animals, such as crabs, starfish, and dab fish that led to different negative effects, such as vomiting, loss of motor control, muscle paralysis, and in some cases death [13,87]. Subsequential analyses found that the crabs, starfish, and dab fish from the beaches presented high concentrations of PSTs, with concentrations 18 times higher than those permitted by the European Union (i.e., over 14,000 µg STX eq/kg found in one starfish sample) [13]. Overall, Turner and coworkers, using LC-FLD analysis, reported that the PSTs present in beach samples were three analogues: dicarbamoyl saxitoxin (dsSTX), saxitoxin (STX), and gonyautotoxins 5 (GTX5) [87].

Another marine toxin that has been reported as causing problems in companion animals is palytoxin. Palytoxin is a marine toxin produced by several soft corals and some dinoflagellates, cyanobacteria, mollusks, crabs, and fish [13]. Bates and coworkers have highlighted that the majority of the cases of companion animal exposure are connected with zoanthid corals, which are grown in saltwater aquaria [13]. Indeed, animals can encounter these corals during the practice of creating or cleaning aquariums by their owners, since a lot of “coral powder” is produced, aerosolized, and inhaled. Most of the reports of exposure to palytoxin from zoanthid coral are connected to an indirect aerial exposure after the cleaning of the aquarium [13]. However, there are some cases reporting the direct exposure of dogs to palytoxins, through licking or chewing rocks in which the zoanthid coral is present [13]. Palytoxin affects the sodium–potassium ATPase pumps, inducing the influx of sodium and the efflux of potassium, causing membrane depolarization. Dogs exposed to palytoxins present negative effects, such as vomiting, disorientation, motor injury, respiratory distress, and in some cases death [13].

### 2.7. Sawfly Larvae

The European birch sawfly, *Arge pullata,* is an insect from the order Hymenoptera, the phylum Arthropoda, and the family Argidae. Argidae is a large sawfly family, with over 800 species [88]. These flies feed on deciduous trees; *Arge pullata* feed, especially, on the leaves of the European white birch (*Betula pendula*). This plant is widely distributed in Europe, through Siberia, China to Japan [89].

Lophyrotomin is the main active compound in sawfly larvae, and it is an octapeptide with hepatotoxic effects [90,91,92,93,94,95], with poisoning cases reported in sheep, cattle, and dogs in Denmark [89,91,92]. Lophyrotomin produced by larvae is used for protection purposes, as it is noted that birds do not feed on them [88,93]. The toxicity is retained in the dead larvae, as well [93]. The larvae feed during late summer to early autumn on the leaves of the birch tree, then they abandon the trees and spin a cocoon in the litter layer, where they spend the winter [88]. During this migration, the larvae spend time in the ground vegetation [88]. Large numbers of larvae are present on the grass, in cases of heavy infestation of the birch tree [93]. While on the ground vegetation, they are occasionally consumed by grazing animals or curious pets [88,93].

The larvae are pale green, with the middle instars showing yellowish areas on both ends and small black dorsal spots and large lateral blotches on the trunk. The larvae have a head, cervical sclerite, legs, and spiracles. Late, and final, instars are about 22 mm long and have a white–gray trunk above the spiracles, and the ventral regions and the anterior part of the prothorax are yellow [89].

The toxic peptides are components of many bioactive metabolites in different microbial, plant, and animal species [94]. As mentioned, lophyrotomin is the principal toxin in sawfly larvae [90,93], and it is an octapeptide with four D-amino acids [94], with a molecular weight of 1039 Da [96]. Other active ingredients include a similar octapeptide and three heptapeptides: pergidin, 4-valinepergidin, and dephosphorylated pergidin [94]. These active ingredients are stable to enzymatic degradation due to the presence of unnatural D-amino acids and potentially remain in animal tissues for a long time [93].

Lophyrotomin is highly toxic to vertebrates, having an intraperitoneal LD50 of 2 mg/kg in mice [97]. Each larvae contains approximately 50 µg of lophyrotomin [94]. The exact mechanism of action of the toxin is unknown. Previous studies have shown that the cellular uptake of lophyrotomin by the hepatocytes is facilitated by the bile acid transport system, in a similar manner to mycrocystin-LR [96], and causes acute centrilobular necrosis. Lophyrotomin also acts on cellular membranes due to its special structure, namely the presence of both hydrophilic and hydrophobic groups at both ends of the molecule [93].

Lophyrotomin has been involved in mass poisoning episodes that caused the death of livestock (hundreds of cattle, sheep, goats, and pigs) in various regions of the world, first reported in eastern Australia, then in Denmark and South America [91,95]. The disease has an acute evolution and is usually fatal [90]. The most consistent clinical signs in affected livestock are weakness, depression, anorexia, incoordination, and difficulty in rising [91,97]. Clinical signs in cattle include excitation, adoption of a high stepping gait, and aggressiveness toward human beings [93]. Occasionally, icterus and photodermatitis are observed [98]. Aggression is associated with hepatic encephalopathy, which is secondary to acute hepatic necrosis [93,99].

Gross pathological findings include ascites, hemorrhages over the serosal surfaces of the thoracic and abdominal cavities, as well as mottled and enlarged liver. On the cut surface of the liver, bright red depressed irregular areas of centrilobular necrosis are seen. The rumen and the gut may contain many larval fragments [91,98]. The spleen is usually enlarged and hemorrhagic [99]. Histologically, in the liver, diffuse, massive centrilobular coagulative necrosis and hemorrhage in the centrilobular areas are seen. In the spleen, depletion of the splenic white pulp is observed and, in the kidneys, renal tubular degeneration is observed [91,99]. The disease was reported in Europe, in Denmark, by Thamsborg et al. [91], who reported that 50 ewes and weaners, from a flock of 250, died after consuming the larvae of *Arge pullata* [91]. Also, in Denmark, a fatal case of sawfly larvae (*Arge pullata*) poisoning was reported in a puppy, by Brummerstedt and coworkers [92].

### 2.8. Scorpions

In Europe, there are approximately 75 species of scorpions identified [100], among which 35 species have been reported in the literature [101]. They belong to four families, namely *Buthidae*, *Euscorpiidae*, *Chactidae*, and *Iuridae* [100,101]. The composition of scorpion venom varies between species [102]. The venom may act on nerve endings of the autonomic system or cause local necrosis [102]. Cardiovascular and pulmonary lesions, leading to pulmonary edema, may ensue due to scorpion envenomation [103].

To the authors’ knowledge, no reports of scorpion sting envenomation of domestic animals in Europe have been published. However, in other continents, there might be evidence that scorpion stings can occur in domestic dogs and cats [102]. *Buthus occitanus* belongs to the *Buthidae* family and is present in Spain and France. It may be the only potentially life-threatening scorpion for humans in Europe, but case reports are scarce [104]. Injections of *B. occitanus* venom in cats, guinea pigs, and rats, were experimentally performed [103]. Direct and indirect cardiovascular effects were reported, including activation of the autonomic system and electrolyte imbalances (hyperkalemia and hypocalcemia). Toxins from the venom also inhibit voltage-gated sodium channel inactivation. The administration of antivenom concurrent with or after the administration of venom was completely or partially protective, respectively [103]. While scorpion sting envenomation seems possible in domestic animals in Europe, further investigations are warranted to study the prevalence and clinical importance, as well as to exhaustively list the scorpion species present in Europe.

### 2.9. Snakes

In Europe, four venomous snakes are commonly reported [105,106]. They belong to the family *Viperidae*, the subfamily of *Viperinae*, and the *Vipera* genus. *V. berus* (European viper or adder) includes three recognized subspecies and expands its habitat from England to much of Europe. *V. aspis* (asp, asp viper, European asp, aspic viper) includes five subspecies, and its habitat includes France, Spain, Switzerland, and Italy. *V. ammodytes* (sand viper, horned viper, long-nose viper), which includes five subspecies, is believed to be the most dangerous European viper, and its habitat includes Italy and southeastern Europe. *V. ursini* (meadow viper, Ursini’s viper, Orsini’s viper, field adder) is an endangered species and its habitat includes southeastern France, Italy, Greece, and eastern Europe [107].

Because all these vipers are poisonous to humans and cause similar clinical signs, it is likely that all domestic animals can be poisoned by any of these species. However, at the time of publication, the European reports on snake envenomation of companion animals or livestock involved *V. berus*, and less commonly *V. aspis* [107]. Their venom contains high molecular weight, complex proteins, with hemo-, myo-, neuro-, and cytotoxicity properties, including towards the endothelium of blood vessels. A specific cardiotoxin was isolated from the venom of *V. berus*, while *V. aspis* venom may have greater neurotoxic properties [11,105,108,109,110,111,112]. The venom may stimulate the release of cytokines. resulting in vasodilation, increased vascular permeability, and edema, which can cause hypovolemia and distributive shock [11].

Small animal envenomation is most frequently reported in the literature, followed by livestock envenomation, including horses, cattle, and sheep [107]. Dog envenomation by vipers seems common in some European countries [11,106,109], while cat envenomation is considered uncommon [111]. Snake bites occurred mostly during the warmer months of the year, but, in the UK, some dog and cat envenomations occurred during the cooler months [108]. Most bites occur in rural or peri-urban outside environments, but livestock may be bitten in indoor pens or stalls [107].

The predilection sites of bites are the muzzle and face in dogs and horses, limbs in cats, or both sites in ruminants [11,107]. Bites on limbs may lead to more severe clinical cases in dogs [111]. Overall, *Vipera* bites caused local swelling and inflammation, with or without systemic signs; for e.g., up to 33% of dogs developed only local clinical signs following adder envenomation in a UK report [107,113]. More specifically, the clinical signs reported in dogs included fang marks, edema, lethargy, pain, vomiting, and diarrhea, which are signs consistent with disseminated intravascular coagulation, tachycardia, hyperthermia, cardiac arrhythmia, and collapse [11,107,109,111]. In some *V. berus* envenomed dogs, cardiotoxicity was evident through increased cardiac troponin I and arrhythmia detected on the ECG [109]; transient liver injuries were detected in some dogs [11]. Envenomation from *V. aspis* may cause more severe symptoms, including hemolytic anemia, respiratory depression, myonecrosis, neuropathy, and acute renal failure, in addition to the listed signs [105,106,112]. A clinical grading was used to describe the severity of the adder envenomation in dogs and was correlated with renal injuries [109]. Tubular injuries were evident in some dogs, but the final diagnosis of these injuries can be challenging to obtain and may require the use of specific biomarkers [110]. The clinical signs of Viperinae envenomation reported in livestock include marked swelling at the site of the bite, potentially leading to airway obstruction with facial bites, blood oozing from fang marks and nostrils, tissue necrosis, bacterial infection, severe pain, ptyalism, dysphagia, depressed mentation, and tachycardia [107]. Mild colic and cardiac arrhythmia were also reported in *V. berus* envenomed horses [108]. It is noteworthy that in non-European countries, pigs and chickens presented clinical signs from the bites of Viperinae [107].

While most animals should recover from snake envenomation, it can be fatal for both companion animals and livestock [107]. However, due to the low number of publications, few mortality rates have been reported. The reported mortality rates from *V. berus* envenomation were from 0.5% to 4% of envenomed dogs [11,108,113], 1 of 3 envenomed cows [107], and from 14 to 43% of envenomed horses [108]. *V. aspis* envenomation may have a higher mortality rate, such as 6% of envenomed dogs in a particular report [106]. A final diagnosis of *V. berus* envenomation, through the confirmation of venom in the blood circulation, was described in a clinical case [111].

### 2.10. Ticks

In 2023, the European Centre for Disease Prevention and Control (ECDC) and the European Food Safety Authority identified the following seven species of ticks in Europe: hard ticks, from the family Ixodidae, namely *Ixodes ricinus*, *I. persulcatus*, *Rhipicephalus sanguineus*, *Dermacentor reticulatus*, *Hyalomma lusitanicum*, *H. marginatum*, and soft ticks, from the family Argasidae, namely *Ornithodorus erraticus* [114].

The mechanism of action of tick paralysis is not fully elucidated, but there was evidence that salivary proteins share similarity with some arachnid venom. One paralytic toxin was characterized in the Australian tick *I. holocyclus*, and the neurotoxicity of some other tick’s toxins were demonstrated [115,116]. Tick toxins interfere with the synthesis and/or release of acetylcholine [2]. The result of this interference is lower motor neuron paresis and paralysis, similar to that induced by the botulinum toxin [2].

Most reports of tick paralysis were in humans, mainly in the US and Australia [115,116]. However, there may be evidence that at least 12 tick species (present in Europe, the Mediterranean region, the Palearctic, or worldwide, regions encompassing the European continent) caused paralysis in domestic animals, including ungulates, dogs, poultry, and other birds, or wild animals, such as seabirds and tortoises [115]. Among these ticks, only three species were indexed by the ECDC. Furthermore, one reference stated that six species of ticks in Europe may cause tick paralysis [116]. Therefore, further research is warranted to exhaustively list the tick species present in Europe, their distribution, and to confirm that domestic animals can be affected by tick paralysis in Europe.

### 2.11. Toads

Many amphibian species are potentially poisonous, but toads have been associated with the majority of clinical cases in animals [2]. There are more than 300 species of true toads (Bufo) found throughout the world. Bufo toads are members of the family Bufonidae, amphibians of the order Anura [117]. Toads are not venomous, as they lack venom apparatus, but they are considered poisonous. Poisonous toads have mucous glands located in their skin, distributed all over their body, which produce a secretion that keeps their skin moist. The toxic substances are produced in the granular glands and the modified mucous glands located on the head, shoulders, and dorsal part of their body [2].

Although there are many different species, only large-sized toads can produce sufficient poison to cause toxicosis in animals; smaller toad species are dangerous if multiple individuals are ingested [2]. The European toad or common toad, *Bufo bufo*, is widely distributed throughout Europe, and is also found in north Africa and north-west Asia [118]. Toad poison is a complex mixture, containing different biogenic amines and steroid derivatives. The main constituents are bufadienolides, the major type being bufadienolide glycosides, which are a type of cardiac glycosides [2,119].

Bufadienolide glycosides inhibit sodium–potassium ATPase pumps in cardiac myocytes, similar to other cardiac glycosides, causing an increased level of calcium inside the cardiomyocytes and consecutive cardiac arrhythmias [2]. Other important constituents of toad poison are bufotenines, which are indolalkylamines (tryptamine derivatives) related to serotonin and 5-hydroxytryptophan with hallucinogenic effects [2,9,120]. Bufotenines, in combination with catecholamines (adrenalin and noradrenalin), are responsible for some of the neurological and gastrointestinal effects of toad poison [2].

Dogs are most commonly involved in toad toxicosis, and mouthing of toads stimulates the release of toxins from the poison glands [2]. Biting, licking, or ingestion of the toad may lead to intoxication [121]. The initial clinical signs are represented by profound, occasionally foamy, salivation, accompanied by strong head shaking, pawing at the mouth, retching, and vomiting, which appear immediately after exposure [2,9]. Hyperemia of the mucous membranes and tachypnea are also common signs. Neurological signs are frequently observed and are represented by convulsions and tremors, ataxia, muscle rigidity, nystagmus, stupor, or coma [2,9]. Cardiac arrhythmias are common consequences of exposure and include bradycardia, sinus tachycardia, and sinus arrhythmias [2,9].

Information about toad venom poisoning in domestic animals in Europe is scarce, with only a few case reports available in the literature. Hernández-Rebollo et al. [118] reported three cases of toad poisoning in southwest Spain. All of the animals presented gastrointestinal, neurological, and cardiac signs with variable severity. The signs observed were apathy, stupor, ventricular arrythmias, and neurological signs, such as ataxia, muscle rigidity, and walking in circles [118]. Scheer et al. [122] reported a fatal case of common toad poisoning in a fox terrier puppy in the Czech Republic, and in poisonings in small dogs in France [4,123]. Between January 2015–March 2019, the Poison Control Centre of Milan (CAV) received three calls about dogs exposed to toad toxins [6]. In addition to domestic animals, toad poisoning was described in zoo animals in Germany [124]. Specifically, Toennes et al. [124] reported the disease in a South American fur seal (*Arctocephalus australis*) in a zoo in Dortmund, Germany.

### 2.12. Other Venomous/Poisonous Species

Other important venomous and poisonous species for which many case reports are published involving humans, but are not yet reported in animals, are *Loxosceles rufescens*, the Mediterranean recluse spider; *Latrodectus tredecimguttatus*, the European black widow; *Cheiracanthium punctorium*, the yellow sac spider, and *Vespa mandarinia*, the Asian giant hornet [125,126].

## 3. Conclusions

This review paper offers an insight into the complex issue of zootoxins and lists the reported causes of zootoxicoses in domestic animals in Europe, where information on this subject is limited and cases are underdiagnosed and underreported, despite being of considerable importance from a health perspective. Even if various poisonous and venomous species are widely distributed throughout Europe, cases have only been reported in a few countries, in which some form of national poison control/reporting system exists. This aspect emphasizes the necessity of a centralized European reporting system, which would allow us to have a better picture about the incidence of different toxicoses, including poisoning with zootoxins across European countries. The most common zootoxicosis in domestic animals involves terrestrial zootoxins produced by the common toad, the fire salamander, the pine processionary caterpillar, and vipers. Other terrestrial zootoxicoses reported or suspected in Europe involved blister beetles, bees, wasps, and sawfly larvae. While scorpions, ticks, centipedes, spiders, and giant hornets may cause zootoxicosis in animals, no cases have yet been reported in Europe. Regarding rare zootoxicoses from marine species, paralytic shellfish toxins and jellyfish were reported sources of intoxication in Europe. However, echinoderm toxins (sea stars, starfish, etc.), mollusk toxins (cone shells, octopus, etc.), or fish toxins (venoms and poisons), may cause zootoxicosis, but have not yet been reported in Europe.

This review will help the veterinary community and animal owners be aware and appropriately respond to emerging toxicological issues associated with exposure to terrestrial and marine zootoxins.

## Figures and Tables

**Table 1:** Taxonomic classification of the venomous and poisonous animal species responsible for poisonings in domestic animals in Europe.

Toxin Source	Phylum	Order	Species Scientific Name	Species Common Name
Terrestrial zootoxins	Arthropoda	Coleoptera	*Meloidae* family *Mylabris variabilis* *Epicauta rufidorsum* *Berberomeloe majalis*	Blister beetles
		Hymenoptera	*Arge pullata*	European birch sawfly
			*Apis mellifera* *Vespula germanica* *Vespula vulgaris*	Honeybee European wasp Common wasp
		Ixodida	*Ixodes ricinus*, *Ixodes persulcatus*, *Rhipicephalus sanguineus*, *Dermacentor reticulatus*, *Hyalomma lusitanicum*, *Hyalomma marginatum* *Ornithodorus erraticus*	Ticks
		Lepidoptera	*Thaumetopoea pityocampa* *Thaumetopoea processionea*	Oak processionary caterpillar Pine processionary caterpillar
		Scolopendromorpha (Subphylum Myriapoda)	*Scolopendra* spp.	Centipede
		Scorpiones	*Buthus occitanus*	Scorpion
	Chordata (Class: Amphibia)	Anura	*Bufo bufo*	Common toad
		Urodela	*Salamandra salamandra*	Fire Salamander
	Chordata (Class: Reptilia)	Squamata (Family Viperidae)	*Vipera berus* *Vipera aspis* *Vipera ammodytes* *Vipera ursini*	European viper European asp Horned viper Meadow viper
Marine toxins	Cnidaria	Zoantharia	Multiple species	Zoanthid coral
		Subphylum: Medusozoa	Multiple species	Jellyfish
	Cyanobacteria	Multiple orders	Multiple species	Multiple species
	Myzozoa			

**Table 2:** Venomous and poisonous animal species and zootoxins responsible for poisonings in Europe.

Common Name	Scientific Name	Main Toxin	Animal Species Affected	Exposure Site	Outcome	Countries
Blister beetles	*Meloidae* family *Mylabris variabilis* *Epicauta rufidorsum* *Berberomeloe majalis*	Cantharidin	Horse	Ingestion	Intense irritation of the skin and the digestive mucous membranes, and urinary tract; myocardial dysfunction; potentially lethal	Slovenia
Honeybee European wasp Common wasp	*Apis mellifera* *Vespula germanica* *Vespula vulgaris*	Melittin, phospholipase A2, apamin and hyaluronidase Peptides, enzymes, amines	Dog, cat, horse (any species)	Skin: lightly furred areas (mainly head area); oral mucosa	Edema, swelling, pain and erythematous plaques at the site of the sting; potentially lethal (multiple stings, anaphylactic reaction)	Switzerland, probably involved in stings in most European countries
Pine processionary caterpillar Oak processionary caterpillar	*Thaumetopoea pityocampa* *Thaumetopoea processionea*	Urticating setae (dual mechanical-toxic effect); Thaumetopoein, the main active compound	Dog and cat	Oral and ocular mucosa, skin (mainly head area)	Oral, cutaneous, and ocular lesions, gastrointestinal and/or airway inflammation; survival rate is high (97–100%)	France, Italy, Spain, Portugal
Centipede	*Scolopendra* spp.	Peptides and proteins acting as neurotoxins, myotoxins, cardiotoxins, as well as pro- and anticoagulant toxins	Dog and cat	Skin: any site (mainly head area); oral mucosa	Local pain, erythema, bruising, and swelling; rarely lethal	Unreported in domestic animals in Europe, centipede–dog/cat interactions reported in France
Fire salamander	*Salamandra salamandra*	Samandarine and samandarone	Dog	Oral exposure: eating, licking, or mouthing of the amphibian	Convulsions, muscle tremors, hypertension, hyperventilation, cyanosis, and cardiac arrhythmias; acute evolution and usually fatal outcome	Italy, Slovenia
Jellyfish	Cnidaria (phylum)	Neurotoxic, cytolytic, hemolytic, and enzymatic (proteases, phospholipases) toxins, including prostaglandins (15R)-PGA2, palytoxin, pseudopterosin, sarcodictyins, and eleutherobin	Dog	Skin: lightly furred areas, such as the face, feet, and abdomen; oral mucosa	Pain, oral irritation and swelling, sialorrhea and minor gastrointestinal signs; rarely lethal	Italy, United Kingdom
Freshwater cyanobacteria and marine dinoflagellates; clams, mussels, geoducks, oysters, and snails accumulate toxins produced by cyanobacteria or dinoflagellates	Cyanobacteria (phylum), *Alexandrium* spp., *Gymnodinium catenatum*	Paralytic shellfish toxins (saxitoxin and its derivatives formed by the addition of sulfo, hydrosulfate, and N-1-hydroxyl groups)	Dog	Ingestion of marine species such as crab, starfish, and dab fish	Vomiting, loss of motor control, muscle paralysis, and in some cases death	United Kingdom
Zoanthid coral	Zoantharia (order)	Palytoxin	Dog	Indirect aerial exposure after the cleaning of the aquarium; licking or chewing rocks in which the zoanthid coral is present	Vomiting, disorientation, motor injury, respiratory distress, and in some cases death	United Kingdom
European birch sawfly	*Arge pullata*	Lophyrotomin	Sheep, cattle, and dogs	Ingestion of larvae	Weakness, depression, anorexia, incoordination; aggression associated with hepatic encephalopathy; acute evolution, usually fatal	Denmark
Scorpion	*Buthus occitanus*	Neurotoxins	Dog and cat	Skin	Cardiovascular and neurological effects, potentially lethal	Unreported in domestic animals in Europe
European viper European asp Horned viper Meadow viper	*Vipera berus* *Vipera aspis* *Vipera ammodytes* *Vipera ursini*	High molecular weight complex proteins, with hemo-, myo-, neuro-, cardio-, and cytotoxicity properties	Dog, cat, horse, cattle, and sheep	Muzzle and face in dogs, and horses, limbs in cats, or both sites in ruminants	Local swelling and inflammation, disseminated intravascular coagulation, cardiotoxicity, neurotoxicity, hemolytic anemia, respiratory depression, myonecrosis, and acute renal failure; potentially lethal	Italy, Spain, France, Switzerland, Sweden, Norway, Finland, Germany, United Kingdom
Ticks	*Ixodes ricinus*, *Ixodes persulcatus*, *Rhipicephalus sanguineus*, *Dermacentor reticulatus*, *Hyalomma lusitanicum*, *Hyalomma marginatum*, *Ornithodorus erraticus*	Neurotoxin	Dogs, cattle, sheep, poultry	Skin: lightly furred areas	Lower motor neuron paresis and paralysis; rarely lethal	Unreported in domestic animals in Europe; anecdotal reports in many countries
Common toad	*Bufo bufo*	Bufadienolides Bufotenines	Dog	Biting, licking, or ingestion of the toad	Salivation, head shaking, pawing at the mouth, retching and vomiting, convulsions and tremors, ataxia, muscle rigidity, nystagmus, stupor or coma, cardiac arrhythmias; potentially lethal	Czech Republic, France, Italy, Spain

**Table 3:** Emerging zootoxins in Europe.

Venomous/Poisonous Animal	Toxin	Domestic Animals Affected
Blister beetles	Cantharidin	Horse
Pine processionary caterpillar Oak processionary caterpillar	Thaumetopoein	Dog and cat
Centipede	Peptides and proteins acting as neurotoxins, myotoxins, cardiotoxins, as well as pro- and anticoagulant toxins	Dog and cat
Jellyfish	Neurotoxic, cytolytic, hemolytic, and enzymatic (proteases, phospholipases) toxins, including prostaglandins (15R)-PGA2, palytoxin, pseudopterosin, sarcodictyins, and eleutherobin	Dog
Prokaryotic freshwater cyanobacteria and eukaryotic marine dinoflagellates; clams, mussels, geoducks, oysters, and snails accumulate toxins produced by cyanobacteria or dinoflagellates	Paralytic shellfish toxins (saxitoxin and its derivatives formed by the addition of sulfo, hydrosulfate, and N-1-hydroxyl groups)	Dog
Zoanthid coral	Palytoxin	Dog

## Data Availability

Not applicable.
